# Sirt3 Mediates the Inhibitory Effect of Adjudin on Astrocyte Activation and Glial Scar Formation following Ischemic Stroke

**DOI:** 10.3389/fphar.2017.00943

**Published:** 2017-12-22

**Authors:** Xiao Yang, Keyi Geng, Jinfan Zhang, Yanshuang Zhang, Jiaxiang Shao, Weiliang Xia

**Affiliations:** State Key Laboratory of Oncogenes and Related Genes, School of Biomedical Engineering, Shanghai Jiao Tong University, Shanghai, China

**Keywords:** ischemic stroke, adjudin, Sirt3, astrocyte activation, functional recovery

## Abstract

In response to stroke-induced injury, astrocytes can be activated and form a scar. Inflammation is an essential component for glial scar formation. Previous study has shown that adjudin, a potential Sirt3 activator, could attenuate lipopolysaccharide (LPS)- and stroke-induced neuroinflammation. To investigate the potential inhibitory effect and mechanism of adjudin on astrocyte activation, we used a transient middle cerebral artery occlusion (tMCAO) model with or without adjudin treatment in wild type (WT) and Sirt3 knockout (KO) mice and performed a wound healing experiment in vitro. Both our *in vivo* and *in vitro* results showed that adjudin reduced astrocyte activation by upregulating Sirt3 expression. In addition, adjudin treatment after stroke promoted functional and neurovascular recovery accompanied with the decreased area of glial scar in WT mice, which was blunted by Sirt3 deficiency. Furthermore, adjudin could increase Foxo3a and inhibit Notch1 signaling pathway via Sirt3. Both the suppression of Foxo3a and overexpression of N1ICD could alleviate the inhibitory effect of adjudin *in vitro* indicating that Sirt3-Foxo3a and Sirt3-Notch1 signaling pathways were involved in the inhibitory effect of adjudin in wound healing experiment.

## Introduction

As a main cell population in the brain, astrocytes participate in lots of key signaling events including anti-oxidant activity, energy transfer, neurotransmitter up-take and recycling, ion homeostasis, trophic factor synthesis and neurovascular coupling (Abeysinghe et al., 2016). In addition to these physiological functions, astrocytes activated by brain injury such as stroke have been reported to show astrogliosis which is characterized as hypertrophic morphology and over-proliferation of astrocytes (Maragakis and Rothstein, 2006). Furthermore, glial scar formed by reactive astrocytes creates a physical barrier for axon regeneration and causes further tissue damage (Barres, 2008; Bao et al., 2012). Therefore, it is necessary to regulate the activation of astrocyte and reduce glial scar formation to overcome the physical barrier and promote neuronal regeneration in response to brain injury.

Proinflammatory cytokines have been shown to mediate astrocyte proliferation (Sriram et al., 2006). After stroke, inflammatory reaction induces the activation and proliferation of astrocytes to form a glial scar (Huang et al., 2014; Rodriguez-Grande et al., 2014). Sirtuins are a family of NAD^+^-dependent enzymes, involved in the control of reactive astrogliosis, which have the potential to modulate neurologic disease (Scuderi et al., 2014). Recent studies have shown that Sirt1 could alleviate astrocyte activation via regulating the MAPK pathway activation after traumatic brain injury (Li et al., 2017). As one member of sirtuins, Sirt3 can reduce reactive oxygen species (ROS) by activating forkhead box O3a (Foxo3a) and attenuate palmitate-induced inflammation (Sundaresan et al., 2009; Koyama et al., 2011). Furthermore, Foxo3a can inhibit the inflammatory cytokine-mediated activation of human astrocytes (Cui et al., 2011).

Our previous studies have shown that adjudin, as a potential Sirt3 activator, can inhibit the proliferation of cancer cells *in vitro* and on prostate and lung tumors inoculated in nude mice (Xia and Geng, 2016). In addition, adjudin attenuates lipopolysaccharide (LPS)- and stroke-induced neuroinflammation due to the inhibition of ERK MAPK phosphorylation and NF-κB activation (Shao et al., 2013; Liu et al., 2014). Therefore, we hypothesized that adjudin might have a protective role against glial scar formation in response to brain injury.

To determine the underlying roles of Sirt3 and adjudin in astrocyte activation after stroke, we used primary astrocytes and performed a mouse model of transient middle cerebral artery occlusion (tMCAO) in wildtype (WT) and Sirt3 knockout (KO) mice. Here, we firstly reported that Sirt3 mediated the inhibitory effect of adjudin in astrocyte activation and glial scar formation.

## Materials and methods

### Reagent and animal

DMSO was purchased from Sigma Aldrich (St. Louis, MO, USA). Adjudin was provided by Dr. C Yan Cheng of the Mary M. Wohlford Laboratory, Population Council, New York. WT mice (129/SvImJ) were purchased from Beijing Vital River Laboratory Animal Technology Corporation (Beijing, China) and Sirt3 KO mice (129-Sirt3^tm1.1Fwa^) were obtained from Jackson Laboratories (Bar Harbor, ME). Mice were housed in individually ventilated cages in a specific pathogen free facility, with 12 h daylight/dark cycles at 23°C. Bromodeoxyuridine (BrdU) powder (Sigma-Aldrich, St Louis, MO) was dissolved in 0.9% NaCl in a concentration of 10 mg/mL. BrdU solution was injected intraperitoneally at 50 mg/kg twice a day for 7 consecutive days 2 and 5 weeks after tMCAO.

### Surgical procedures

Animal procedures were performed according to the protocol approved by the Institutional Animal Care and Use Committee of Shanghai Jiao Tong University, Shanghai, China. Adult male mice weighing 25–30 g (8- to 10-week-old) were used in the study. Mice were assigned to the adjudin-treated group, the DMSO-treated group or the sham group. The surgical procedure of MCAO was described previously (Yang et al., 1994). Briefly, mice were anesthetized with ketamine (100 mg/kg) and xylazine (10 mg/kg) intraperitoneally. Mice were placed supinely on a heating pad (RWD Life Science, Shenzhen, China), which maintains body temperature at 37.0 ± 0.5°C. The left common carotid artery (CCA), the external carotid artery (ECA) and the internal carotid artery (ICA) were isolated. 6-0 suture (Dermalon, 1741-11, Covidien, OH, USA) coated with silicone was introduced into the ECA stump and advanced from the ICA to the opening of the middle cerebral artery (MCA) until a slight resistance was felt. At this moment, the tip of the suture was located in the anterior cerebral artery (ACA). All procedures were performed under an operating microscope (Leica, Wetzlar, Germany). The success of occlusion was characterized as the reduction of cerebral blood flow (CBF) down to 10% of baseline, which was verified by a laser Doppler flowmeter (Moor Instruments, Devon, England). The ICA was occluded for 1.5 h, followed by the removal of the suture to allow reperfusion. The sham group underwent the same procedure without suture insertion. Mice were injected with adjudin (50 mg/kg, DMSO stock dissolved in corn oil at a dilution of 1:10) or DMSO (DMSO dissolved in corn oil at a dilution of 1:10) intraperitoneally every 2 days performed 2 days after reperfusion until sacrifice.

### Measurement of infarct volume

Mice were sacrificed 4 days after surgery; brain tissue was immediately collected and frozen into pre-chilled isopentane. The brain was cut into 20-μm-thick coronal sections. The cryosections were immersed in 0.1% cresyl violet for 30 min and rinsed in distilled water for 10 min. The infarct area in each section was calculated by the ImageJ software using the following formula: contralateral hemisphere area (mm^2^) - ipsilateral undamaged area (mm^2^). Infarct volume between two adjacent sections was calculated by this formula:

$$\frac{1}{3}\times \text{h}\times \left({\text{S}}_{1}+{\text{S}}_{2}+\sqrt{{\text{S}}_{1}\times {\text{S}}_{2}}\right)$$

where S1 and S2 are the infarct areas of the two sections, and h is the two sections' distance. The total infarct volume is the sum of all infarct volume from each pair of adjacent sections.

### GFAP immunostaining

Five serial cryosections, spaced 200-μm apart (0.5 mm frontier and 0.5 mm posterior from the ischemic core), were selected from each mouse. Brain cryosections (20 μm in thickness) were fixed with methanol at −20°C for about 10 min and washed with PBS. Next, these sections were blocked with 10% BSA for 1 h. After washed with PBS, they were incubated with the primary antibodies (mouse anti-GFAP antibody, 1:300 dilution, BD Biosciences, San Jose, CA, USA) overnight at 4°C. Sections were incubated with Alexa-488-conjugated secondary antibody (1:200 dilution, Life Technologies) for 1 h, and washed with PBS. Nuclei were then stained with 4,6-diamidino-2-phenylindole (DAPI) (1:500 dilution, Beyotime Institute of Biotechnology, China). A confocal laser-scanning microscope was used to obtain confocal microscopic images (Leica TCS SP5 II, Germany).

### NeuN/BrdU, and CD31/BrdU double immunostaining

Brains were post-fixed for 24 h followed by 48 h of immersion in 30% sucrose in PBS and immediately frozen, and then sectioned using a freezing microtome (Leica, Solms, Germany). A thickness of 20-μm coronal sections was cut. Floating coronal sections were collected in antigen protective solution, which involves 20% glycol, 30% glycerol and 50% PBS. Sections were first treated with 2 mol/L HCl for 20 min at 37°C and then neutralized with sodium borate twice each for 10 min. Sections were then treated with 0.3% Triton-X 100 in PBS for 15 min, blocked by 10% BSA, incubated with NeuN (1:100; Millipore), or CD31 (1:200; R&D) antibody at 4°C overnight. Finally, the sections were incubated with secondary antibodies (1:500; Thermo Fisher) for 60 min at room temperature. Stained sections were mounted after rinsing.

### Primary astrocyte cell culture

Primary astrocyte cultures were prepared from neocortical tissues of 1-day-old mice. Briefly, the cerebral hemispheres were isolated. The meninges, the hippocampus, basal ganglia, and olfactory bulb were carefully removed. Neocortical tissues were minced and incubated into 0.25% trypsin for 15 min at 37°C. Cell suspension was centrifuged at 1,000 rpm for 5 min and washed with PBS. Then the cells were resuspended in DMEM containing 10% fetal bovine serum and 100 units · ml^−1^/100 μg · ml^−1^ penicillin/streptomycin at 37°C in a humidified incubator with 5% CO_2_. After 24-h cell culture, the medium was replaced.

### C8-D1A cell culture

Mouse astrocytic cell line C8-D1A was obtained from EK-Bioscience company (Shanghai, China) and cultured in DMEM (HyClone) supplemented with 10% fetal bovine serum (HyClone), 100 I.U. Penicillin and 100 mg/mL Streptomycin (Invitrogen) at 37°C in a humidified 5% CO_2_ incubator.

### Wound healing assay

To gauge the ability of wound healing, nonclosed gap area was tested. 100,000 cells were seeded into 12-well plates per well and after growing to ~100% confluence, the confluent cell monolayer was scraped with a pipette tip. The length of gap was measured at 0 and 24 h after scratch. The experiments were performed in triplicate.

### Oxygen-glucose deprivation (OGD)

Astrocytes were incubated in DMEM containing 100 units·ml^−1^/100 μg·ml^−1^ penicillin/streptomycin and 10% fetal bovine serum, and the cells washed twice and incubated in glucose-free DMEM. The cultures were transferred into a chamber filled with a gas mixture of 95% N2/5% CO_2_ at 37°C. Three hours after OGD, astrocytes were incubated in DMEM containing 100 units·ml^−1^/100 μg·ml^−1^ penicillin/streptomycin and 10% fetal bovine serum and reintroduced to the regular atmospheric oxygen level for an additional 24 h. In each experiment, cultures exposed to OGD were compared with normoxic controls supplied with DMEM containing glucose and maintained in standard incubation conditions.

### Cell proliferation assay

Cell proliferation was determined by using Cell Counting Kit-8 (Yeasen, Shanghai, China). Briefly, ~10,000 cells were seeded into a well of 96-well plates. After treatment, CCK-8 solution was added to each well at the dilution of 1:10. After incubation for 1-3 h, the absorbance at 450 nm was measured by microplate reader (Synergy2, BioTek).

### Western blot

Cells were lysed with RIPA buffer (Millipore, Temecula, CA, USA) containing protease and phosphatase inhibitor cocktail (1:100) with 2 mM PMSF. The protein concentration was determined by using bicinchoninic acid (BCA) protein assay kit (Pierce, Rockford, IL, USA). 30 μg of total protein was used for this assay. Protein was separated by 10% SDS-polyacrylamide gel electrophoresis (SDS-PAGE), and then it was transferred to 0.45 μm Nitrocellulose Membrane (Millipore, CA, USA).

After the overnight incubation with primary antibodies at 4°C, the membrane is hybridized with secondary antibody (1:4,000 dilution, Epitomics, China) at room temperature for 1 h. Protein signals were detected by ECL system. The primary antibodies were listed as follows: Sirt3 (1:1,000 dilution, Cell Signaling Technology, Danvers, USA); Notch1 (1:1,000 dilution, Cell Signaling Technology, Danvers, USA); Foxo3a (1:1,000, Cell Signaling Technology, Danvers, USA); β-Tubulin (1:1,000 dilution, Abcam, Hong Kong); N1ICD (1:1,000 dilution, Abcam, Hong Kong).

### Real-time PCR

Total RNA was isolated from cells by RNAiso Plus (TaKaRa, Dalian, China), 1 μg of total RNA was used to synthesize first strand cDNA with PrimeScript RT reagent kit (TaKaRa). Real-time quantitative PCR was carried out on ABI 7900HT with the use of SYBR Premix Ex Taq (TaKaRa) on the basis of the following protocol: 95 for 30 s, 40 cycles consisting of 95 for °C °C 5 s and 60°C for 30 s, 95°C for 15 s, 60°C for 1 min, and 95°C for 15 s. Primers were as follows: GFAP (sense 5′-CGGAGACGCATCACCTCTG-3′ and anti-sense 5′-AGGGAGTGGAGGAGTCATTCG-3′); Sirt3 (sense 5′-ATCCCGGACTTCAGATCCCC-3′ and anti-sense 5′-CAACATGAAAAAGGGCTTGGG-3′); Rplp0 (sense 5′-AGATTCGGGATATGCTGTTGGC-3′ and anti-sense 5′-TCGGGTCCTAGACCAGTGTTC-3′). Data were analyzed by using the comparative threshold cycle (Ct) method, and results were expressed as fold difference normalized to Rplp0.

### Plasmid and siRNA transfection

Cells were seeded to 24-well plate or 6-well plate at the concentration of 40,000 cells or 200,000 cells per well respectively. With 24-h cell culture, transfection was then performed using Lipofectamine 3000 (Invitrogen) according to the manufacturer's protocols. siRNA sequences were as follows: Foxo3a siRNA (sense: 5′-GAACGUUGUUGGUUUGAAUdT-3′, anti-sense: 5′-AUUCAAACCAACAACGUUCdTdT-3′).

### Luciferase reporter assay

In order to study the transcriptional level of N1ICD, C8-D1A were co-transfected by using the Lipofectamine 3000 reagent (Invitrogen) with 500 ng of the 4xCSL luciferase reporter (Figure S1) together with 500 ng of N1ICD plasmid and 10 ng of pRL-TK reference plasmid each well. After 48-h transfection, cells were treated with adjudin for 24 h and extracts were harvested. The number of replicates per experiment was 3 independently (*n* = 3), luciferase activity was measured using the reporter assay system (Promega, Madison, WI, USA). The N1ICD plasmid (#41730) and 4xCSL luciferase reporter (#41726) were purchased from Addgene. pRL-TK reference plasmid was provided by Pro. Gao Wei-Qiang (Shanghai jiaotong university, Shanghai, China).

### Rotarod test

To examine balance and coordination deficits, the rotarod test was introduced (Li et al., 2002). The mice were placed on the accelerating rotarod whose speed was increased from 5 to 40 rpm. The time that mice were on the rotarod was measured. The animals were trained prior to MCAO to obtain stable baselines. The test was performed on 2, 7, 14, and 35 days post-tMCAO surgery.

### Statistical analysis

The data were given as mean ± standard deviation. Comparisons between two groups were analyzed by Student's *t*-tests. Data comparisons between multiple groups were analyzed by one-way ANOVAs with Tukey's *post-hoc* tests. *P*-values less than 0.05 considered statistically significant. All statistical analyses were performed using GraphPad Prism version 3.05 (GraphPad Software, Inc., La Jolla, CA).

## Results

### Adjudin inhibited astrocyte activation following ischemic stroke

**Figure 1:**
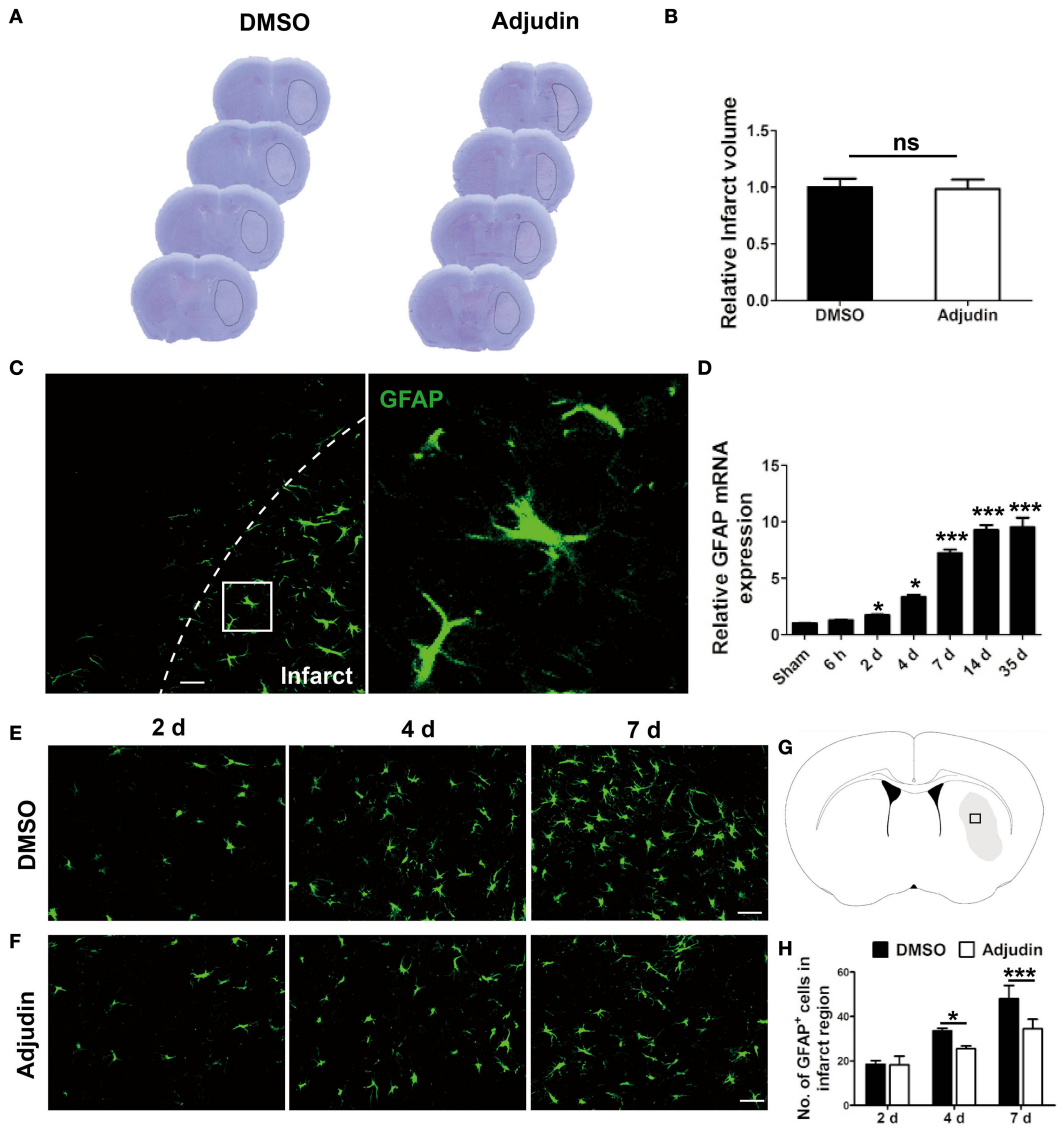
Adjudin inhibited stroke-induced astrocyte activation. Mice were treated with either DMSO or adjudin 2 days after transient middle cerebral artery occlusion (tMCAO). **(A)** Cresyl violet staining of brain sections from mice which were performed tMCAO and were administrated with either DMSO or adjudin 4 days after reperfusion. Line indicates infarct area. **(B)** Quantification of the infarct volumes. **(C)** The changes in astrocyte morphology were detected by immunofluorescence *in vivo*. The GFAP-positive astrocytes were green. **(D)** RT-PCR showed the GFAP expression after stroke. one-way ANOVA: ^*^P < 0.05, ^***^P < 0.001 vs. the sham group (*n* = 4 mice per group), the sham group: control without tMCAO. **(E,F)** The number of GFAP^+^ cells decreased significantly in the adjudin-treated group compared with the DMSO-treated group at 4 and 7 days after tMCAO. **(G)** Hollow box represent the picture that in the infarct region of ischemic brain. **(H)** Quantification of GFAP^+^ cells. one-way ANOVA: ^*^P < 0.05, ^***^P < 0.001 (*n* = 5 mice per group). Bars represent the mean ± SEM. Scale bar, **(B)** 50 μm; **(E)** 50 μm; **(F)** 50 μm.

Our previous studies have shown adjudin protected against cerebral ischemia reperfusion and reduced infarct volume during acute phase of stroke (Liu et al., 2014). To further investigate the role of adjudin following chronic ischemic stroke, we performed adjudin administration 2 days after tMCAO. No significant differences were observed in infarct volume 4 days after ischemic stroke (Figures 1A,B). Our results showed upregulation GFAP of accompanied by hypertrophy of astrocytes in infarct region (Figure 1C). In addition, we detected the mRNA level of GFAP in infarct region, and found that GFAP expression increased significantly at different time beginning at 2 days after tMCAO (Figure 1D). To investigate the role of adjudin in astrocyte activation, we performed immunostaining for GFAP at different times (Figures 1E–G). The results showed that adjudin could significantly reduce the number of GFAP^+^ cell both at 4 and 7 days after stroke, indicating that astrocyte activation could be attenuated by adjudin (Figure 1H).

### Adjudin inhibited astrocyte activation *in vitro*

**Figure 2:**
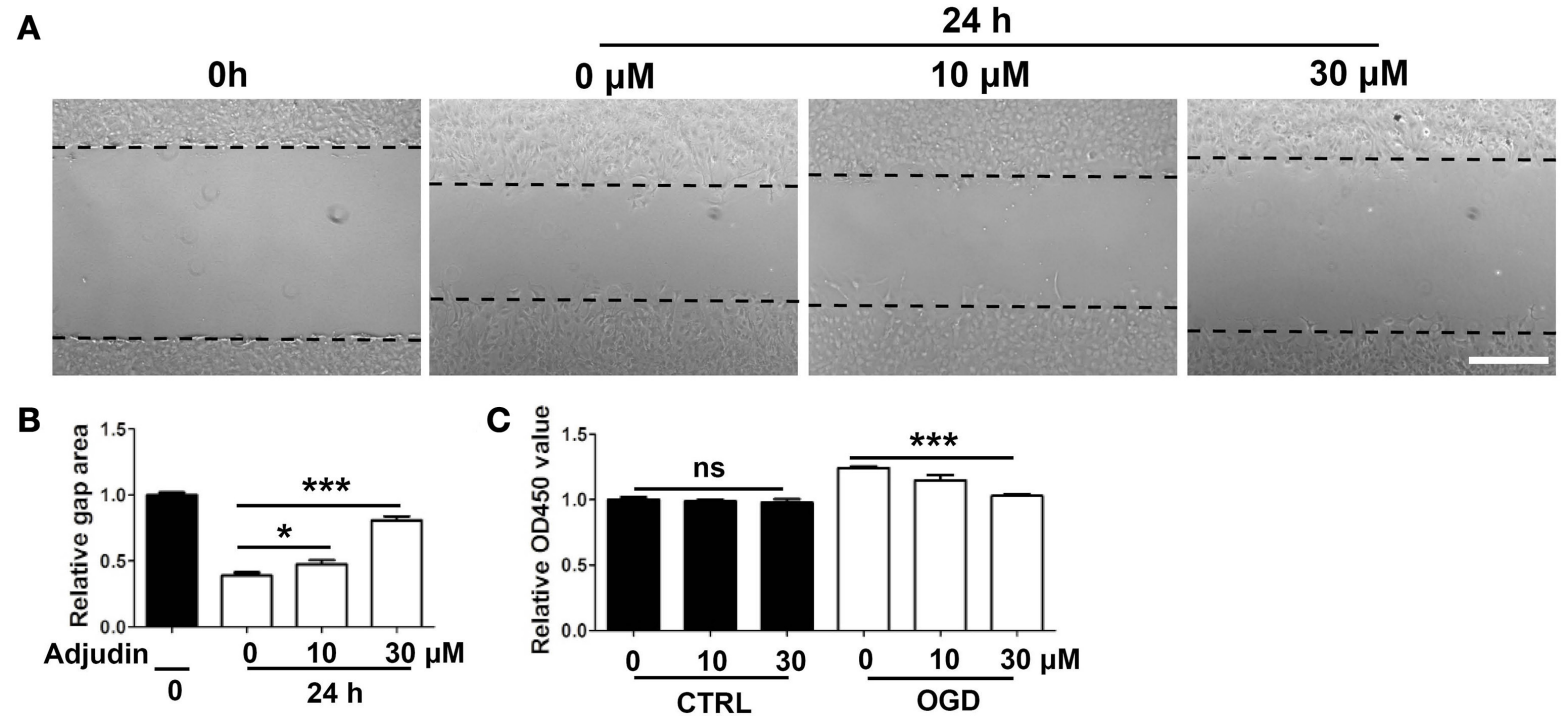
Adjudin inhibited astrocyte activation *in vitro*. **(A)** Effect of adjudin on wound healing in primary astrocytes. Dash line indicated gap area. **(B)** The quantification of gap area showed adjudin could significantly attenuate the wound healing 24 h after scratch. **(C)** The quantification the number of astrocytes showed that adjudin could significantly inhibited OGD-induced activation and proliferation 24 h reperfusion. one-way ANOVA: ^*^P < 0.05, ^***^P < 0.001, ns: no significance. Bars represent the mean ± SEM.

To further confirm the role of adjudin in astrocyte activation, we isolated primary astrocytes and performed wound healing experiments *in vitro*. The results showed that adjudin inhibited the scratch closure, as shown by a larger cell-free area observed 24 h after the wound (Figures 2A,B). Previous studies indicated that the astrocytes presented the characteristics *in vivo* after conditioned OGD (Wang et al., 2012). We found that conditioned OGD (3 h) could induce astrocytes activation and proliferation, as assessed by CCK-8 at 24 h after reperfusion (Figure 2C). Furthermore, we found that adjudin could inhibit astrocyte proliferation induced by OGD, and no differences were detected under normal condition (Figure 2C).

### Sirt3-Foxo3a signaling pathway was involved in the inhibitory effect of adjudin on astrocyte activation

**Figure 3:**
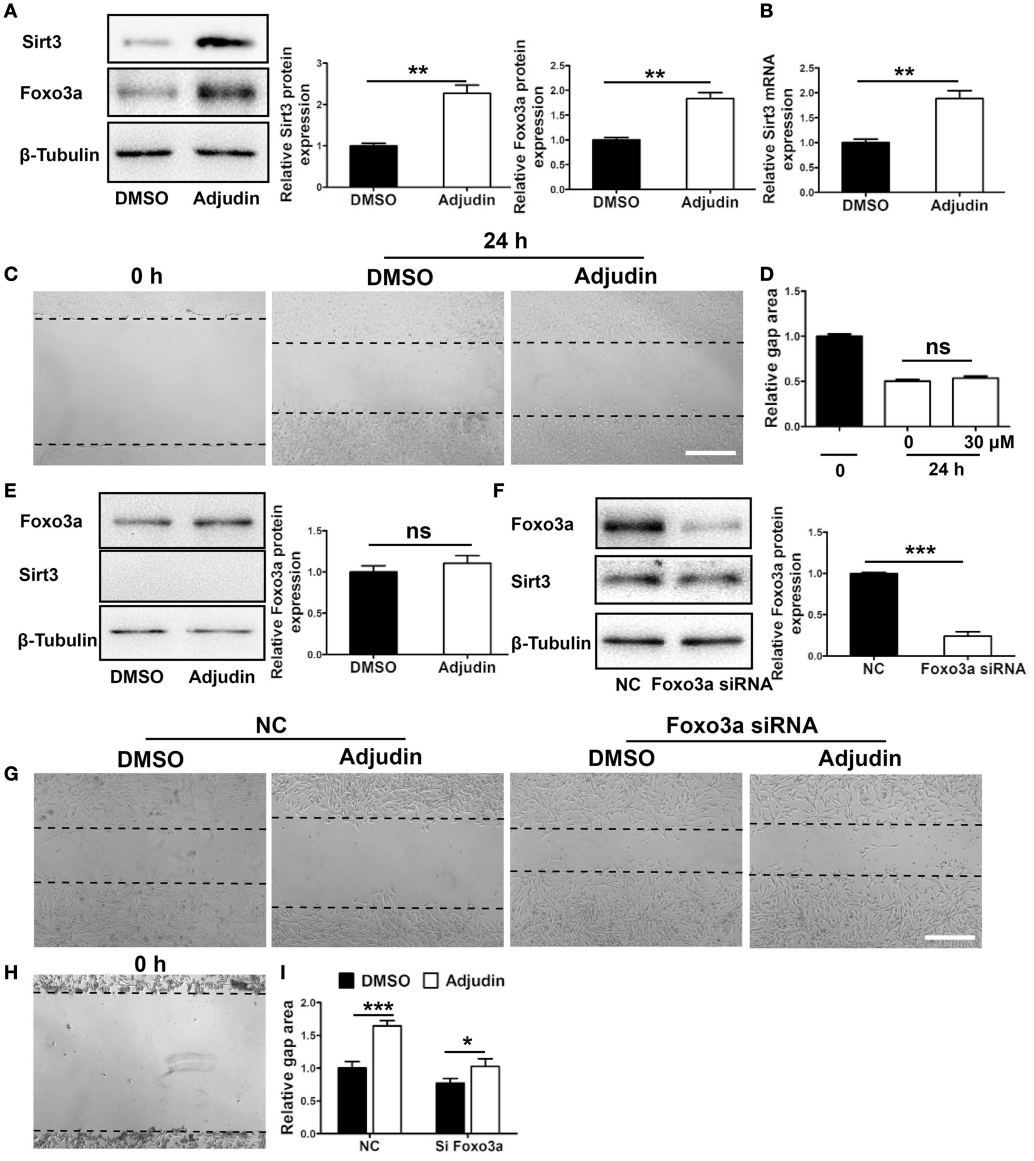
Sirt3-Foxo3a signaling pathway mediated the inhibitory effect of adjudin on wound healing *in vitro***. (A)** Western blot showed that Sirt3 and Foxo3a protein levels were significantly upregulated by adjudin in primary astrocytes. **(B)** The mRNA level of Sirt3 was also upregulated by adjudin *in vitro*. **(C)** Adjudin couldn't inhibit wound healing in Sirt3 KO astrocytes. **(D)** The quantification of gap area in Sirt3 KO astrocytes. **(E)** Adjudin did not increase Foxo3a expression in Sirt3 KO astrocytes. **(F)** Western blot showed Foxo3a protein level was significantly decreased in FOXO3a siRNA-treated C8-D1A cells compared with NC group. *t*-test: ^**^P < 0.01, ^***^P < 0.001, ns: no significance. Bars represent the mean ± SEM. Scale bar, (C) 500 μm. **(G)** Wound healing experiments were performed in C8-D1A cells, and the area of gaps were observed 24 h after scratch. **(H)** The gap area of C8-D1A at 0 h after scratch. **(I)** The quantification of gap area in C8-D1A cells showed Foxo3a deficiency attenuated the inhibitory effect of adjudin. one-way ANOVA: ^*^P < 0.05, ^***^P < 0.001, ns: no significance. Bars represent the mean ± SEM. Scale bar, **(G)** 500 μm.

Our results showed that Sirt3 could be significantly upregulated by adjudin at both protein and mRNA level (Figures 3A,B). To investigate whether Sirt3 mediated the inhibitory effect of adjudin in astrocyte activation, we isolated primary astrocytes from Sirt3 KO mice. We found that Sirt3 deficiency abolished the inhibitory effect of adjudin assessed by wound healing experiment (Figures 3C,D). In addition, Foxo3a could also be upregulated by adjudin, which was attenuated by Sirt3 deficiency (Figures 3A,E). To confirm whether Foxo3a was involved in Sirt3-mediated effect of adjudin, we transfected Foxo3a siRNA in C8-D1A astrocytic cell lines (Figure 3F). The inhibitory effect of adjudin in wound healing can be blunted in Si Foxo3a cells although significant difference between two experiment groups still exist. (Figures 3G–I). These results indicated that Sirt3-Foxo3a signaling pathway might mediate the inhibitory effect of adjudin on astrocyte activation *in vitro*.

### Notch1 signaling pathway was involved in Sirt3-mediated inhibitory effect of adjudin on astrocyte activation

**Figure 4:**
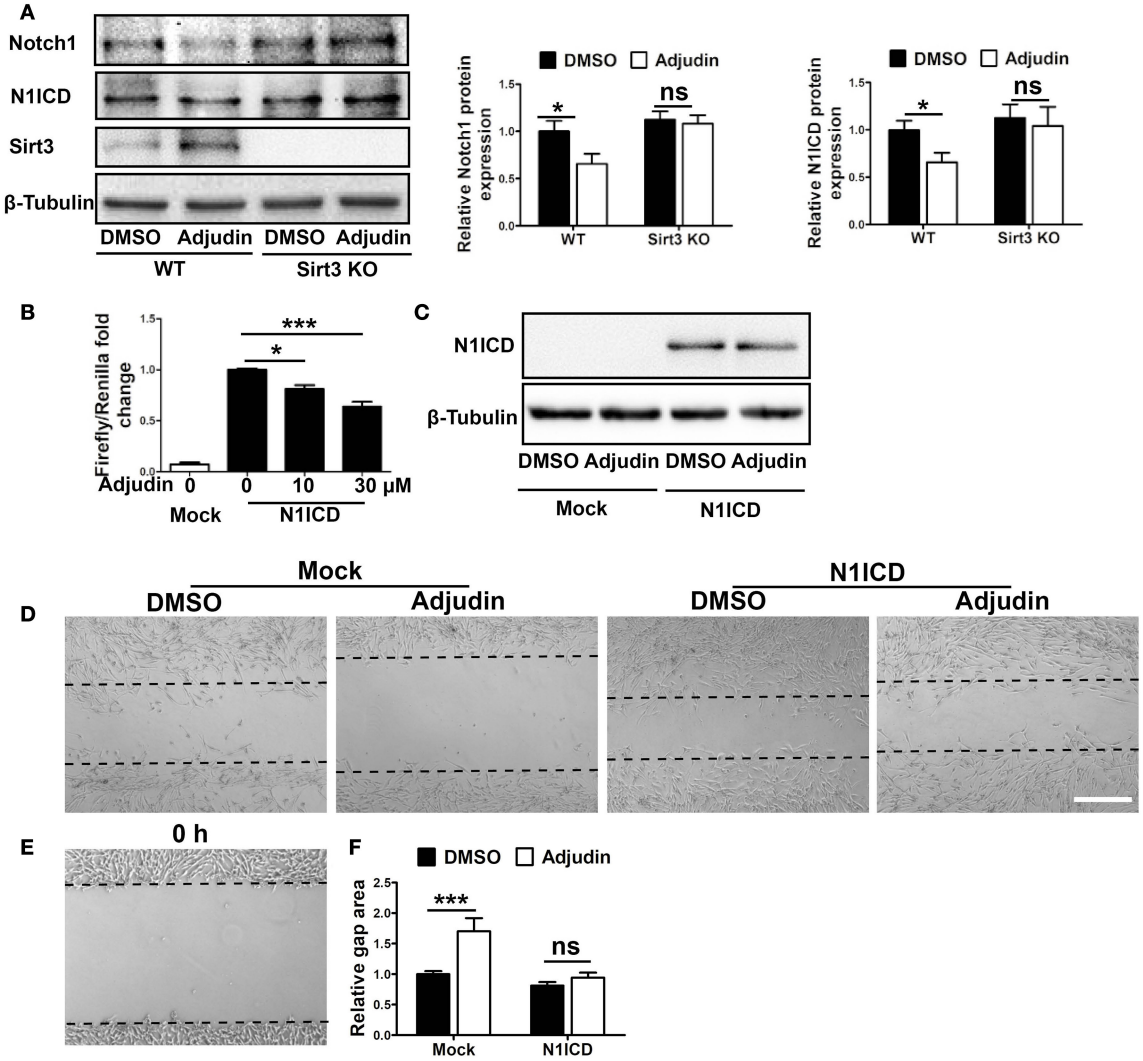
Notch1 signaling pathway was involved in Sirt3-mediated inhibitory effect of adjudin on wound healing *in vitro*. **(A)** Western blot showed Notch1 and N1ICD protein levels were decreased by adjudin in WT astrocytes, and no significantly differences were observed in Sirt3 KO astrocytes. **(B)** Luciferase experiment showed adjudin significantly inhibited the transcriptional activity of N1ICD. **(C)** To study the role of Notch1 signaling pathway, we transfected N1ICD plasmid into C8-D1A cells. Western blot indicated N1ICD protein level. **(D)** We performed wound healing experiments performed in C8-D1A cells, and we observed gap area 24 h after scratch. **(E)** The gap area of C8-D1A at 0 h after scratch. **(F)** The gap area of C8-D1A at 0 h after scratch. The quantification of gap area in C8-D1A cells showed N1ICD overexpression blunted the inhibitory of adjudin. one-way ANOVA: ^*^P < 0.05, ^***^P < 0.001, ns: no significance. Bars represent the mean ± SEM. Scale bar, **(D)** 500 μm.

Previous study have shown that Notch1 could regulate the proliferation of reactive astrocytes in the peri-infarct region after stroke (Shimada et al., 2011). We found that Notch1 and active form of N1ICD (Notch1 intracellular domain) protein levels decreased significantly in adjudin treated astrocytes, which was blunted by Sirt3 deficiency (Figure 4A). To explore transcriptional activity of N1ICD, we used 4xCSL (CBF-1–SuH–LAG-1) luciferase reporter which could reflect the activity of N1ICD-CSL-MAML transcription complex. We transfected N1ICD plasmid, 4xCSL luciferase reporter and pRL-TK reference plasmid to C8-D1A cell, and detected the activity of luciferase 24 h later. The result showed that adjudin could reduce the transcriptional activity of CSL, indicating adjudin could inhibit the downstream pathways of Notch1 (Figure 4B). To demonstrate the role of Notch1 signaling pathway in the inhibitory effect of adjudin, we transfected N1ICD plasmid in C8-D1A cells (Figure 4C). Wound healing experiment showed that N1ICD overexpression blunted the inhibitory effect of adjudin (Figures 4D–F).

### Sirt3 mediated the neuroprotective role of adjudin in ischemic stroke

**Figure 5:**
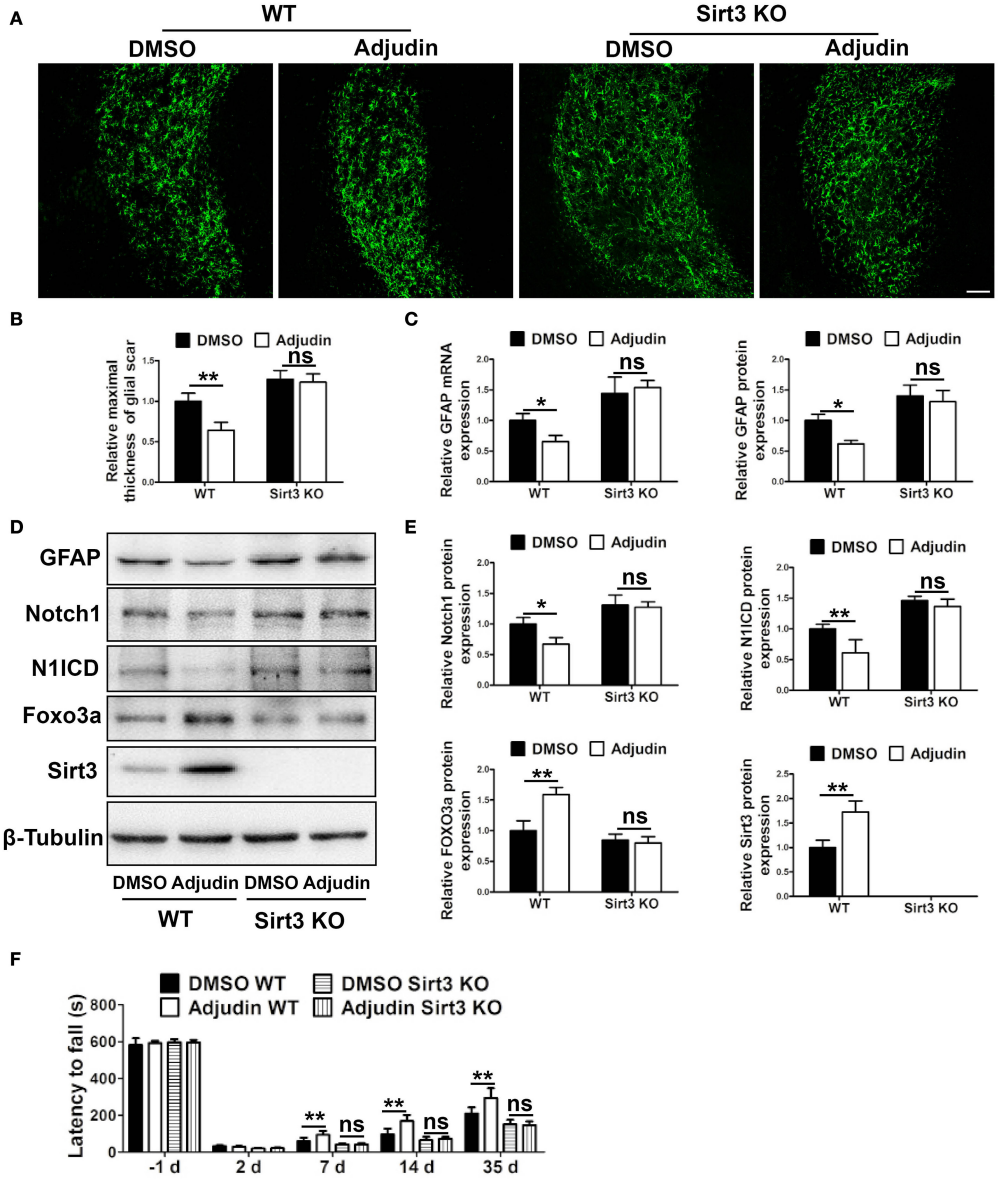
Sirt3 mediated the inhibitory effect of adjudin on glial scar formation following ischemic stroke. Mice were treated with either DMSO or adjudin 2 days after tMCAO. **(A)** The formation of glial scar were inhibited by adjudin in WT mice 35 days after tMCAO, and Sirt3 deficiency blunted the effect of adjudin. **(B)** The quantification of the maximal thickness of glial scar. **(C)** The quantification of GFAP protein and mRNA expression. **(D)** Western blot showed adjudin increased Sirt3 and Foxo3a protein levels and decreased Notch1 and N1ICD protein levels in WT mice 35 days after tMCAO, which were blunted by Sirt3 deficiency. **(E)** The quantification of protein expression. **(F)** Rotarod test showed adjudin increased latency time in WT mice, and Sirt3 deficiency blunted the effect. One-way ANOVA: ^*^P < 0.05, ^**^P < 0.01, ns: no significance (*n* = 5 mice per group). Bars represent the mean ± SEM. Scale bar, **(A)** 100 μm.

Strategies to overcome inhibitory molecules in the scar formation have been indicated to promote functional recovery (Fitch and Silver, 2008). To further study the potential involvement of adjudin in astrogliosis and scar formation, we performed tMCAO model in WT mice. GFAP immunostaining showed glial scar was narrower at 35 days after tMCAO in the adjudin-treated mice compared with that in the DMSO-treated mice, which was abolished in Sirt3 KO mice (Figures 5A,B). Similar changes were detected both in GFAP protein and mRNA levels (Figures 5C,D). Additionally, Western blot analysis showed that Sirt3 and Foxo3a were upregulated and Notch1 signaling was inhibited in adjudin-treated WT mice, as anticipated, Sirt3 deficiency also blunted this effect (Figures 5D,E). Furthermore, the rotarod test showed adjudin-treated mice performed better than the DMSO-treated mice beginning 7 days after tMCAO. However, we did not observe the neuroprotective role in Sirt3 KO mice (Figure 5F). Above results indicated glial scar played a detrimental role in functional recovery following chronic stroke.

### Adjudin promotes neurovascular recovery after ischemic stroke

**Figure 6:**
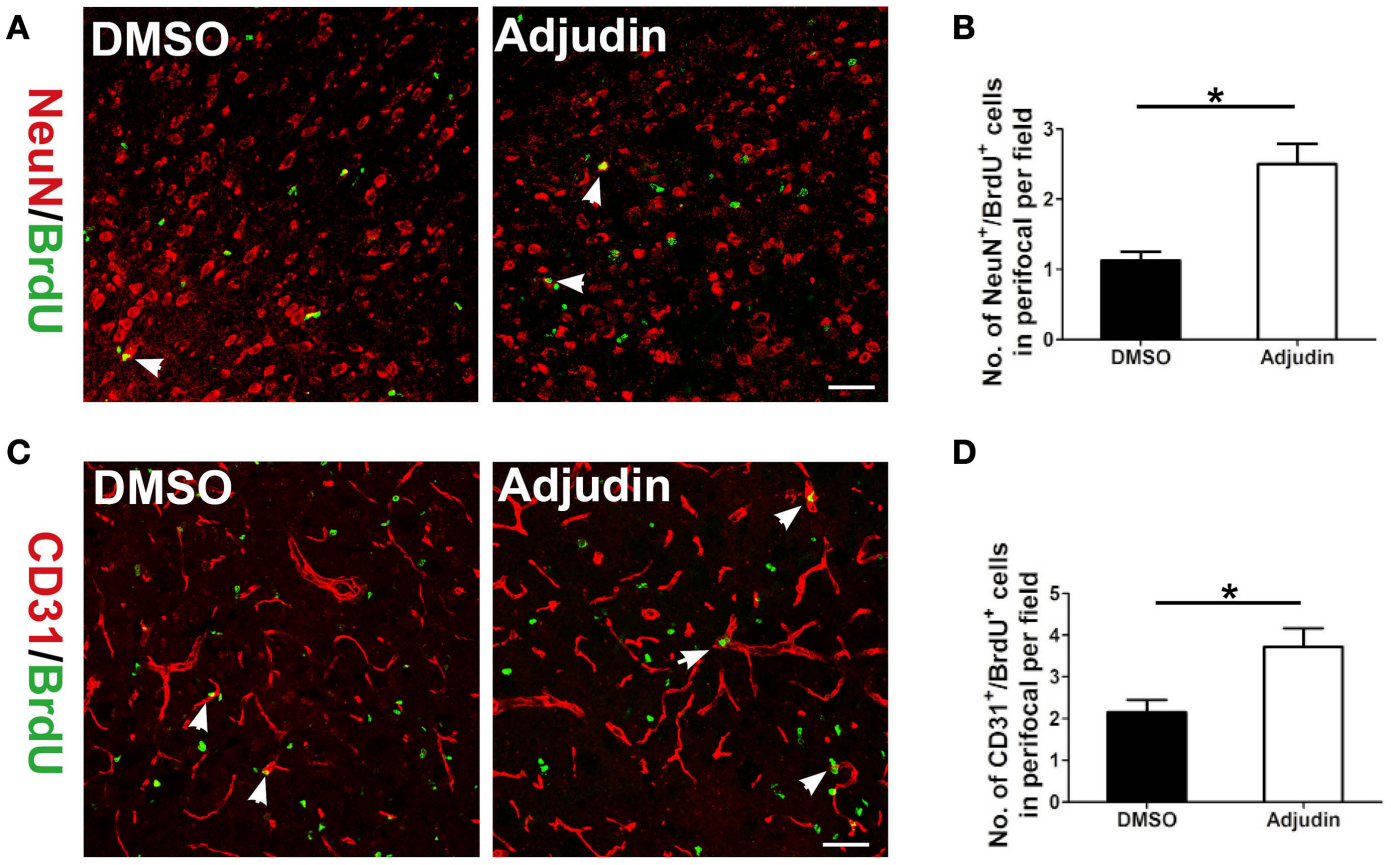
Adjudin promotes neurovascular recovery following ischemic stroke. **(A)** NeuN+/BrdU+ cells were counted in perifocal region of ischemic brain, and **(B)** quantification of NeuN+/BrdU+ cells at 35 days after tMCAO (*n* = 4 per group). **(C)** CD31+/BrdU+ cells were counted in perifocal region of ischemic brain, and **(D)** quantification of CD31+/BrdU+ cells at 35 days after tMCAO (*n* = 4 per group). *t*-test: ^*^P < 0.05. Bars represent the mean ± SEM. Scale bar, **(A)** 50 μm; **(C)** 50 μm.

Neurogenesis and angiogenesis are beneficial for the functional recovery after stroke (Li et al., 2014). To further confirm the role of adjudin in functional recovery following chronic ischemic stroke, we performed double immunostaining for NeuN/BrdU and CD31/BrdU. Compared with the control group, adjudin-treated mice displayed significantly greater numbers of NeuN^+/^BrdU^+^ and CD31^+^/BrdU^+^ cells at 35 days after tMCAO in the perifocal striatum region (Figures 6A–D), indicating that adjudin promoted neurogenesis and angiogenesis from ischemic stroke induced impairments.

## Discussion

**Figure 7:**
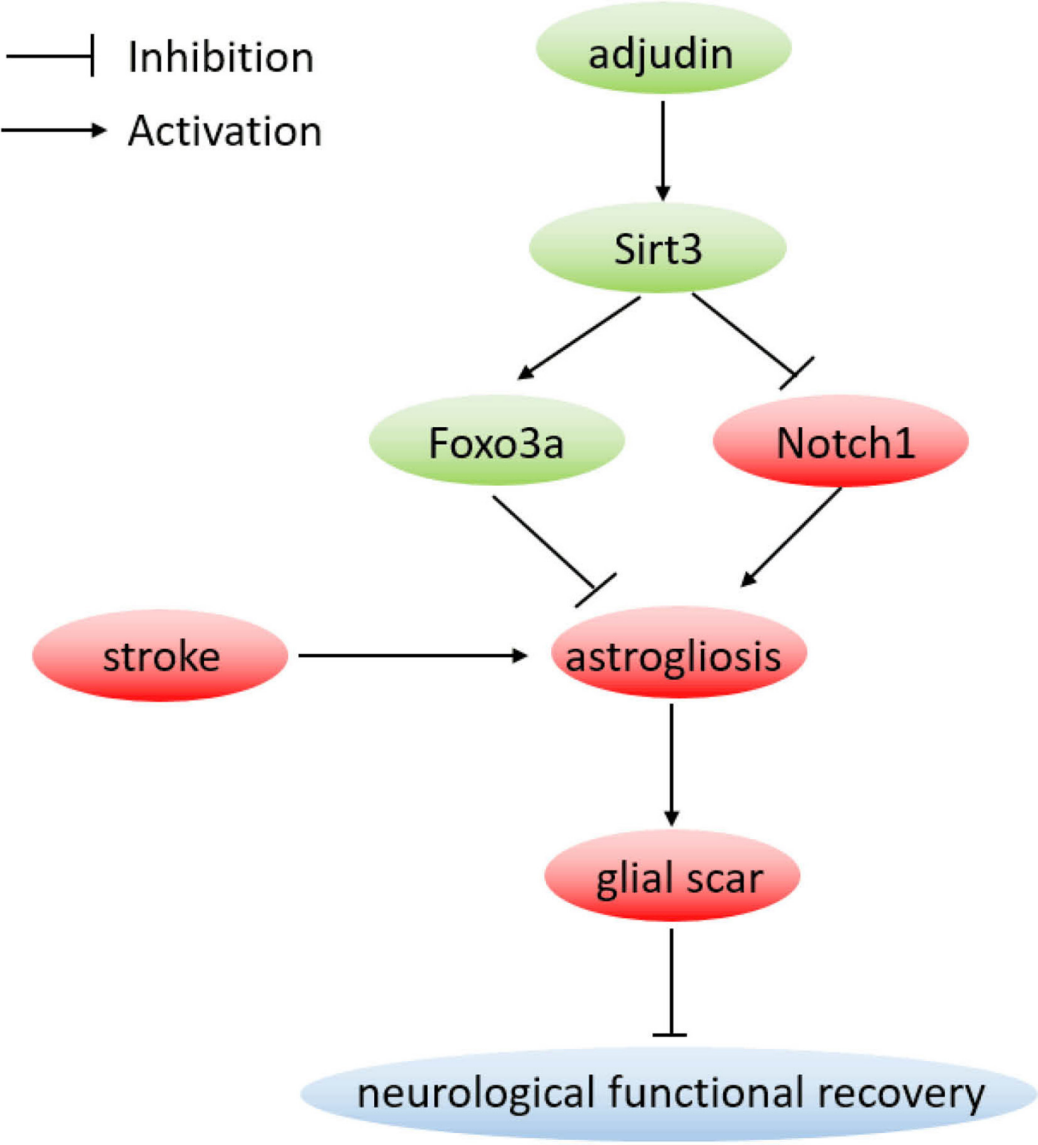
Proposed neuroprotective mechanism by adjudin. Adjudin might inhibit astrocyte activation which could form a scar following stroke by increasing Sirt3 and Foxo3a expression, and inhibiting Notch1 signaling pathway. Furthermore, Foxo3a and Notch1 signaling pathways were regulated by adjudin might via Sirt3.

After stroke, astrocytes change their morphology, proliferate, and migrate to the injury sites to form a scar (Sofroniew, 2009). In the present study, wound starch and oxygen glucose deprivation triggered the activation and proliferation of astrocytes. Our results showed that adjudin could inhibit astrocyte activation both *in vitro* and *in vivo* and Sirt3 was proved to play a crucial role in the neuroprotective function of adjudin. Furthermore, we reported that Sirt3-Foxo3a and Sirt3-Notch1 signaling pathways mediated the inhibitory effect of adjudin against astrocyte activation and glial scar formation following ischemic stroke (Figure 7).

Globally, stroke is the second main cause of death and a leading cause of long disability. Neuroinflammation after ischemia-induced cerebral injury is known to be a key factor that contributes to glial scar formation (Jin et al., 2010) which has been shown to inhibit nerve growth and regarded as a barrier to neuronal repair (Ridet et al., 1997; Jeong et al., 2012). Our previous studies have shown that adjudin can decrease infarct volume and improve behavioral outcome via inhibiting neuroinflammation during the acute phase of ischemic stroke (Liu et al., 2014). In the current study, our data showed that the glial scar was inhibited by adjudin along with improved neurological function during the recovery phase of ischemic stroke. Since infarct size would influence the degree of astrocyte activation and scar formation following injury (Bao et al., 2012), we delayed adjudin treatment to reduce the beneficial effect of adjudin in acute phase. Although recent studies provide compelling evidence that glial scar can exert both beneficial and detrimental effect in a variety of disease processes (Anderson et al., 2016; Robel, 2016), our results indicated that glial scar plays a detrimental role in neurological function recovery following ischemic stroke.

Accumulating evidences indicate that sirtuins are related to diverse neurologic diseases including stroke and served as potential therapeutic targets in the clinical environment (Hernández-Jiménez et al., 2013; Jeśko et al., 2017). In addition, previous studies have shown that both Sirt1 activator and Sirt2-selective inhibitor were able to attenuate the activation of astrocyte as well as the production of proinflammatory cytokines (Scuderi et al., 2014). Sirt3 can be upregulated by adjudin and plays crucial roles in suppressing multiple pathologies including inflammatory responses (Lombard and Zwaans, 2014; Liu et al., 2015). Our results showed that Sirt3 deficiency attenuated the inhibitory effect of adjudin on primary astrocytes wound healing. Furthermore, Sirt3 was demonstrated to be a crucial mediator of adjudin's inhibitory effect in the formation of glial scar and neuroprotective role following ischemic stroke.

The activation of astrocyte and the formation of glial scar involve multiple signaling pathways (Koyama, 2014). Notch1 has been reported as a key factor required for reactive astrocyte activation in the infarct region as well as worsening brain damage and functional outcome after stroke (Arumugam et al., 2006; Shimada et al., 2011; LeComte et al., 2015). Recent studies have shown that Sirt3 could downregulate Notch1 in human gastric cancer, and Notch1 overexpression attenuated the inhibitory effect of Sirt3 on the proliferation of tumor cells (Wang et al., 2015). In addition, our previous studies showed that Notch1 signaling pathway related protein expression were upregulated in Sirt3 KO NSCs compared with WT group (Figure S2). We found Notch1 signaling pathway could be inhibited by adjudin accompanied with increased Sirt3 both *in vivo* and *in vitro*. The rescue experiment proved that Notch1 signaling might interact with Sirt3 and play an important role in the activation of astrocyte. Furthermore, our results showed that adjudin's inhibitory effect in wound healing can be blunted in N1ICD overexpression cells. One possible explanation of our observation is that although adjudin can inhibit the transcription activity of N1ICD, its expression level and transcriptional activity are still very high, which was able to exhibit effect in wound healing.

Foxo3a is also an important mediator for astrogliosis, negatively regulating astrocyte proliferation and reducing cytokine-mediated astrocyte proliferation (Cui et al., 2011). Sirt3 regulates Foxo3a-mediated mitochondrial biogenesis during hypoxia (Tseng et al., 2013). In addition, Sirt3-Foxo3a pathway mediates the neuroprotective effect of ketones in ischemic stroke (Yin et al., 2015). Both *in vivo* and *in vitro*, we found Foxo3a was significantly upregulated by adjudin. Sirt3 deficiency reduced the expression of Foxo3a that was enhanced by adjudin, indicating that Sirt3 functions as an upstream regulator of Foxo3a. Furthermore, knocking down Foxo3a blunted the inhibitory effect of adjudin in wound healing experiment, supporting the view that Sirt3-Foxo3a pathway contributes to the inhibitory effect of adjudin on astrocyte activation.

Neurogenesis and angiogenesis are beneficial for functional recovery. Overactive astrocytes which could form a scar adjacent to the stroke lesion breaks neurovascular coupling in structurally intact nerve. Furthermore, glial scar is not only a physical barrier to neurons trying to connect with vascular but also generates inhibitory molecules preventing axon regrowth (Abeysinghe et al., 2016). Therefore, inhibiting astrogliosis astrocytes to further reduce the glial scar is an important target for neurovascular recovery (Cai et al., 2017). Our results showed that adjudin could promote neurogenesis and angiogenesis following ischemic stroke which might be partly due to the reduction of glial scar area.

## Conclusion

This study investigated the inhibitory effect of adjudin on astrocyte activation. We found that adjudin treatment effectively decreased glial scar area and promoted functional recovery following ischemic stroke. Moreover, we provided evidences to reveal novel Sirt3-Foxo3a and Sirt3-Notch1 pathways as the underlying mechanism.

## Author contributions

WX was responsible for the conception and design of the study conceived the project, coordinated the study, revising it critically for important intellectual content, final approval of the version to be submitted, drafted and revised the manuscript. XY designed the study, or acquisition of data, or analysis and interpretation of data, drafted, revised the article critically for important intellectual content designed experiments, performed animal experimental procedures, rotarod test, immunofluorescent staining, real-time PCR, Western blot, cell culture, plasmid and siRNA transfection, wound scratch, luciferase reporter assay, and analyzed the data, and drafted the manuscript. KG was involved in study design, article drafting and revision, data analysis and experiment including conducted cell culture, wound scratch, immunohistological staining, Western blot, plasmid transfection, cell proliferation assay and data analysis. JZ participated in the animal experimental procedures, Western blot and immunofluorescent staining. YZ conducted part of Western blot and real-time PCR. JS assisted in the experimental design, data analysis and manuscript draft. All authors read and approved the final manuscript.

### Conflict of interest statement

The authors declare that the research was conducted in the absence of any commercial or financial relationships that could be construed as a potential conflict of interest.

## Supplementary material

Figure S1 The plasmid profile of 4xCSL-luciferase.

Figure S2 Notch1 signaling pathway related protein expression were upregulated in Sirt3 KO NSCs compared with WT group.

## Abbreviations

MCAO: middle cerebral artery occlusion
WT: wildtype
KO: knockout
LPS: lipopolysaccharide
GFAP: glial fibrillary acidic protein
N1ICD: Notch1 intracellular domain
Foxo3a: forkhead box O3a.
