# Supplementary figures and images for: Sirt3 Mediates the Inhibitory Effect of Adjudin on Astrocyte Activation and Glial Scar Formation following Ischemic Stroke
Source: Front Pharmacol. 2017 Dec 22;8:943. doi: 10.3389/fphar.2017.00943 (PMC5744009; doi:10.3389/fphar.2017.00943)

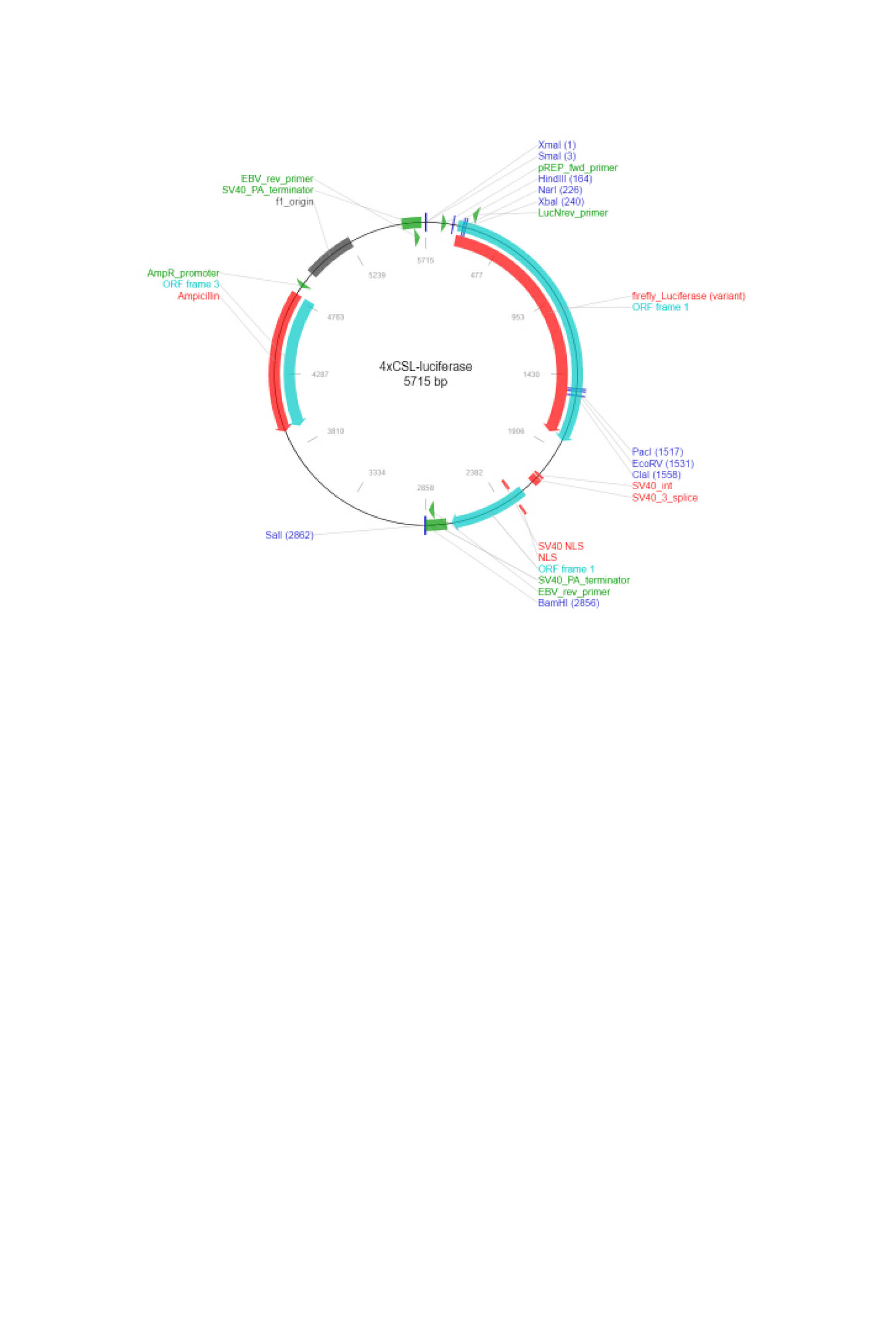

Supplement: Figure S1 — The plasmid profile of 4xCSL-luciferase. [file Image1.JPEG]

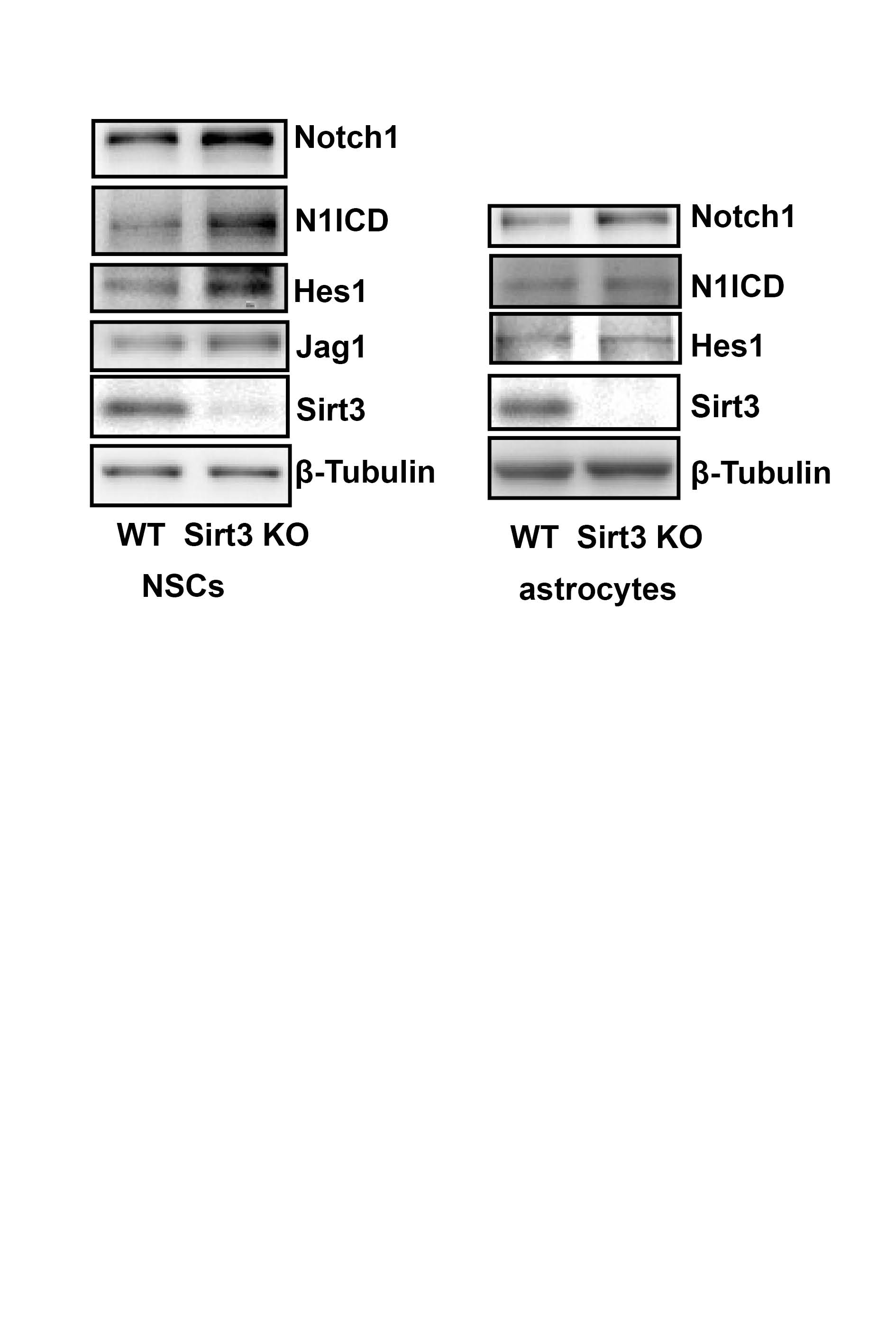

Supplement: Figure S2 — Notch1 signaling pathway related protein expression were upregulated in Sirt3 KO NSCs compared with WT group. [file Image2.JPEG]
